# Differentiation of Remedies

**Published:** 1885-09

**Authors:** P. P. Wells

**Affiliations:** Brooklyn, N. Y.


### HOMŒOPATHIC PHYSICIAN,

A MONTHLY JOURNAL OF MEDICAL SCIENCE.

"If our school ever gives up the strict inductive method of Hahnemann, we are lost, and deserve only to be mentioned as a caricature in the history of medicine."—CONSTANTINE HERING.

Vol. V. SEPTEMBER, 1885. No. 9.

### DIFFERENTIATION OF REMEDIES.

P. P. WELLS, M. D., BROOKLYN, N. Y.

(*Continued from page 270.*)

Symptoms in the arms and hands are often important as guides to the specific remedy for *sicknesses*. Rheumatisms and neuralgias are not seldom chiefly treated in these, and call for one or the other of these drugs. We also find in these, not unfrequently, evidences of important embarrassments at the centre of circulation. These, rightly apprehended and differentiated, will often point to the desired curative. They are also often indicative of conditions of the brain, in sicknesses more or less important, and are always, and in all cases, to be carefully noted and studied, and then they may often become to us great practical lights.

Acon. has *trembling* of arms and hands with *numbness* and *paralysis* of the *left*, which will *not permit* the hand *to rest*. Bell. has *trembling* with *convulsive movements*. Bry. with *jerkings, spasmatic drawings*, and *constant tremblings also of the fingers*.

Acon. has *painful* and *powerless* falling down of the arms. Bell. has arms *numb* and *painful*. *Heaviness, weakness*, especially of the *hands*. *Paralytic weakness*. Paralysis. Bry. has *sensation* of *paralysis* in the *finger*.

Acon. has *coldness* and *loss of sensation* in the *arms*. Bell. has *copious cold* sweat on the *hands*. Bry. has *coldness*, after *heat*, in the *forearm*, and *hands*, in the *morning in bed*.

Acon. has *tearings* in the *arms* from the *shoulder* to the *wrist*

and *fingers*, almost only *when moving*, with *blueness* of the hand *during the pain.* Bell. has *tearing* in the *bones*, and in the *forearm*, and in the bones of the hand, and *paralytic tearing* in the *joints*, and *tearing cutting* in the *muscles.* Bry. has *nervous tearing downward internally, tearing* in the *forearm*, and in the *upper joints* of the *finger.*

Acon. has *bruised pain* in the *shoulder* (and hip) *joints, after sleep,* as from lying on a hard bed, only when moving the parts. Bell. has pain *like a bruise* in the *upper arm.* Bry. has not this pain in the upper extremities.

Acon. has *swelling* of the *muscles* of the *shoulder*, with *bruised pain* when *touched.* Bell. has *swelling* of *hands* and *arms* with *scarlet redness.* Bry. has *shining red* rheumatic *swelling* of the *shoulder joint* and *upper arm*, with *shooting, tearing*, or *tension* when *moving the part.*

Acon. has *stitch* sometimes *drawing* in the *shoulder* and *upper arm.* Bry. has *stitch* in the *upper arm* when raising it.

Bell. has *pressing, tearing* in the *shoulder, running suddenly down the arm*, especially severe at *night, relieved* by pressure; *increased* by motion; Bry. has *drawing* like a *thread* through the *arms* to the *fingers.*

Bell. has frequent *stretching* and *twisting;* arms *numb* and *painful; heaviness, weakness*, especially of the hands; *paralytic weakness; paralysis; crawling, running; not* relieved by *rubbing;* neither Acon. nor Bry. have analogies of these symptoms; Bell. has also *cramps, convulsive movements, jerkings, flexion* and *extension* movements; Bry. has *twitching* and *jerking* in the *deltoid muscle;* Bell. has *rheumatic pains* with *crawling*, also *paralytic pressing* and *drawing*, and *tearing*, and *rigidity* with *tearing* and sense of *shortening* in the *night*, three o'clock A. M.; *drawing* and *jerking* in the *muscles* of the *upper arm; paralytic pressure* in the muscles.

Bry. has *pressing* on the *right* shoulder, with shooting to the fingers when taking a *deep inspiration;* and also *pain* like a *sprain* in the *joint* when *raising* the *arms; pressure* on the *bones* in the *evening*, hindering *sleep;* Acon. has *drawing* in the *elbow joint;* Bell., *paralytic drawing* and *stitch* in the *left joint; cutting* when *walking;* in the hand, as if water were running through the veins; pimples beneath the joint; also *dark red* with *stitch* and *excoriation* when *touched;* Bry. has *swelling* of the *joint; stitch* in the *joint*, especially when *bending* it, with *drawing* as far as the *hand; red rash, shooting* and crawling.

In the *forearm*, Acon. has *heaviness* and *loss of power*, as far as the fingers, with *numb tingling* (asleep) when *grasping; pain* in the *forearm* as if from a *severe blow; drawing* (*tearing, shooting*) also *in the hands;* increased by *motion; paralytic* sensation in *right forearm* and *hand,* especially when *writing, removed* by *strong motion;* Bell. has *shooting, cutting* in the *forearm.*

Acon. has *spasmodic, contractive pains;* sometimes with *stitch* in *hands* and *fingers; tearing* and *paralytic drawing* in the *wrist joint; numbness, icy coldness,* and *insensibility* of *one hand;* cool *sweat* on the *palm* of the hands; *swelling* of the *hands* with *frequent cough* and normal appetite; *drawing, jerking, paralytic,* and *sprained pains* in the *thumb;* when *bending* the *fingers* severe *stitch* in the *wrist* and to the *elbow; crawling pain* in the *fingers,* also when *writing.*

Bell. has *shooting, pressing* pains in the *bones of the hand; sensation* of *rigidity* in *hands* and *fingers;* can only *turn* the *hand* by *starts* toward the shoulder, as if from lack of *moisture in the joints;* small, red *spots* on the back of the hands; *drawing pains* in the *fingers;* easy *cracking* of the joints; *stiffness* of the *joints* with pains when *bending;* the *ends* of the *fingers* pain as if *squeezed;* shooting *outward* in the *finger ends* when *grasping,* with *chill of the body; pain* as if from *ulceration* in the *end* of the *middle finger; inflamed vesicles* on the *fingers; pustules* about the *nails* of the *forefinger.*

Bry. has *crawling* in *hands,* as if asleep; *heaviness* with *stitch* in the joints; *weakness* which does not permit a *firm grasp, shooting* in the *wrist* when becoming *warm;* pain from every motion of the *joint* as if *dislocated* or *sprained;* sensation of *numbness* in the *palm; inflammation* of the *back* of the hand, about midnight, with *burning; shooting* in the hands and fingers and in the muscles *when writing; jerking* of the *fingers* when *moving* them; *hot pale swelling* of the *little finger,* with *shooting* when *pressed* on and when *moving; pain* as of an ulcer in the root of the *little finger* with *pimples* between the thumb and *forefinger,* with *shooting* when *touched.*

The remarks on the importance and relations of symptoms of the upper extremities are equally applicable to those of the lower. In addition it may be remarked that some of the neuralgic affections of the lower limbs, by the severity of the sufferings they cause and the extreme obstinacy of their resistance to the action of palliatives and all other means resorted to for cure, except that of the *true specific,* give greater importance to these symptoms, as it is only by a right study and understanding of them that this specific can be found, which, being found, the cure is assured. In the prosecution of this study we shall find:

Acon. has *paralytic weakness* of *thighs* and *legs* after sitting. Bell. *paralytic tension*, as if *wrenched*, when *walking*. Bry. has *weak* and *unsteady legs*, when *going down-stairs; paralysis* of *legs*.

Acon. has *tensive pressure* in the *thighs*, with *great weakness* when *walking; loss of power* in the *hip-joint*, with *staggering gait*, and *intolerable crushing* pain, especially after *lying down* and *sleeping; numbness* and *paralysis* of the *left leg; unsteadiness* of the *knees*, which *crack* and *stagger* when *walking*.

Bell. has *heaviness* when *walking* with *stiffness* of *the knees;* also with flow of *yellow mucus* from the *nose* and *thirst; weakness*, with *drawing pain; paralytic drawing; paralysis* with *lying down; vertigo, anxiety, nausea*, and *trembling;* in the thighs, *heaviness*—also when *sitting*, but especially when *walking* with *stiffness*.

Bry. has *weakness* with *staggering*, especially when *going up-stairs*, and also *stiffness*, as if from *spasm*, mornings in bed.

Acon. has *drawing, jerking, tearing* in the *knees, ankles*, and *tendo Achillis*.

Bell. has *excoriating* pain on the *inner* surface of the thigh; *shooting*, or *jerking, tearing, cutting* when *sitting; drawing* in the *left* and *pressure* on the *right; shootings* as if from *knives, after eating; throbbing* in the left, *shaking, tingling* in the *right*, when *sitting;* pain in the *hip*, with *burning, shooting* in the *joint*, worst at night and worse from *touch;* pain in the *left*, with *limping;* pain in *left hip* when *lying on the right; cold* sensation on *right hip-joint; stiffness* after *sitting; cramp pains* in *gluteal muscles*, with *tension* when *stooping*.

Bry. has *stitch* in the *hip* as if from *knives*; frightful *stitch* from a *false step*, with *throbbing* in *repose*, and *pain* from *touch; painful jerks* or *blows*, when *lying* or *sitting, better* when *walking;* shootings from the *hip* or *buttock* to the *knee* or *foot*—also with *universal sweat* and intolerance of *touch* or *motion; drawings* in *legs; tearing* from *moving; drawings* as from the *commencement* of the *catamenia; bruised pain*, with *hammering throbbing* when *sitting; itching* also about the *hips*.

Acon. has *heaviness* of the *lower limbs; numbness* and *tingling* of *legs* and *feet*.

Bell. has *bruised pain* with *tearing* in the *joints*, with *stitching gnawing* to the *bones, relieved* by *walking; worse* when *sitting;* with necessity for *constant movement* of the *leg;* inclination to *turning* and *stretching* the *legs*.

In the *knees* Bell. has *severe pain; squeezing* also in the *bend* with *pressure; tightness* when *moving*, as if the *tendons* were *too*

*short; stitch* in the *bend*—also under the *patella* (with *pressure*); *jerking* of the *muscles*—also in the *bend; trembling* of the *knees;* sensation as if the *knees* would *give way*, especially when *walking* or *going up* or *down stairs.*

Bry. has *in the knees—weakness,* with *staggering* and *cracking* when *walking; bruised patella* and pain when *going down-stairs,* as if they *would break; cramps* when *sitting* and *nights* when lying down; *stitch* when *moving* or *walking,* also with *drawing,* as far as the *calf; tensive, painful stiffness;* rheumatic, *shining red* swelling,* with *stitches; tearing* as far as the *shin bone; burning* in the *right, itching* in the *bend,* with *sweat* on the spot *nights;* dry *eruption* in the *bend* and *itching* in the *bend,* in the *evening becoming red* and *smarting* after *scratching; pustules under* the *knee,* with pain and *stitches* when *touched.*

In the *leg* Bell. has intolerable pain, which compels *stretching it out; heaviness,* also *drawing* or *trembling,* or with *stiffness,* as if from *growing,* or in the *right* when laying it *over* the *left; weariness,* also when *going up-stairs,* especially in the *calves,* or *paralytic stitch* in the *leg, upward* as far as the *knee;* also with *external crawling; cutting; drawing,* as far as to the *thighs, loins,* and *shoulders; tearing,* also *pressing* and *burning,* as far as the *bend* of the *knee; squeezing,* as if *wedged,* with *raging* (*toben*), especially at *night,* diminished by the *legs hanging down;* on the *shin pressure* when *standing; tearing,* also *pressing; tearing,* with *pressing asunder.*

Bry. has *in the leg—weakness,* especially when *beginning* to *walk* and when *standing; drawing,* especially in the *bones* or in the *calf,* with *sweat; bruised* pain in the *calf,* especially when *moving* the *leg* or when *feeling* it, with *numbness* in *repose; jerking* in the *leg* at *night,* also in the *daytime,* like *electric shocks; tearing, jerking* in the *shin bone; stitching, tearing, tension,* especially in the *calf* or from the *feet* to the *bend* of the *knee,* also with *shining red swelling; swelling* of the *legs* without *redness,* also *rapid swelling; foul ulcers* on the *leg.*

Bell. has *in the calf—shootings* from *below upward; cramps* evenings *in bed* while *bending the leg.*

Bry. has *cramps* in the *calf, feet, back of the feet,* and *heels,* especially at *night* when *lying down, better* from *moving; cramps* in the *calf* in the *morning;* nocturnal *tearings* in the *back* of the *foot;* pain like a *sprain* or as if from a *false step,* especially when *stepping.*

---

* The *red,* rheumatic swellings, which call for Bry., are almost always *shining* also. This is quite a characteristic of the swelling of this drug.

Acon. has pain in the *ankle*, with *despair* and *fear of death; coldness* of the feet *to the ankles*, with *sweat* on the *soles; coldness*, especially of the *toes;* sensation in *callous bunches* on the feet, as if they were *girt* with a *band* mornings; intolerable pain in the *ankle, relieved* by *external pressure*.

Bell. has *boring* and *tearing* in the *tendo* Achillis; in *the feet, crawling; tension* in the *sole*, also in the *joints* when *walking* in the *open air;* pain in the *meta-tarsal bones* when *walking* and *bending the foot inward; tearing* in the *meta-tarsal bones* of the *great toe* or in the *sole* with *stitch; shootings* in the *soles* and on back of *left foot* when *sitting; boring* and *digging* in the *soles;* cramps in the *soles, evening in bed*, when *drawing up the knees; drawing, running* about the *ankles;* severe or *gnawing itching* on the *feet* and *backs* of *the feet; heat* in the *feet; burning* in the *soles;* sweat on the feet when sitting.

Bry. has *shootings* in the *feet*, also especially *nights* or *mornings* in the *heels* or when *stepping*, especially in the *soles*, with *tension* of the joints, also when *lying down* intolerable *tension* and *shooting; heavy, numb* sensation in the *soles*, especially when *stepping*, as if *tense* and *swollen; tension* in the *joints* of the *feet*, especially when *moving* or in the back of the feet when sitting, also in the *evening* as if the feet were *swollen; hot, inflammatory swelling* of the *feet*, with *redness* and *bruised* pain when *extending* it; *tension* when *stepping* and pain like *ulceration* when *feeling it; gouty swelling* of the *feet; in the toes; bruised* pain *in the balls* of the *left; shootings*, especially in the *balls* of the *great toes*, also with *pressure* and pain as if *frozen*, or especially when *sitting*, less when *walking*, also *evenings* with great sensation of *heat* therein; *corns*, with *pressing pain* when *stepping* and in *repose;* as if *raw* or *burning; stitching* when *touched* and *better* when *pressed on*.

It was our purpose when we entered on this differentiation to give an example of study of our materia medica by the process of individualization, and this because it is by this process only that the specific character of drug action can be reached, and that by this same process only can the specific character of any disease which we may meet be disclosed and thus to impress the fact on the mind, if we might, that the *simile* in which the law of relationship of curatives to sicknesses is found is only in these specific characteristics. This is to be sought and found only by examination of these characteristics of the drug and sick phenomena in their *elementary* individuality. By this analysis and comparison of these two classes of phenomena, and only by this, can any treatment of the sick or any prescription for their relief be *homœopathic,* except as a result of *accident*—a lucky blunder.

Homœopathy is in its nature a philosophy of individualization. Without this, Homœopathy *is not.* We bring this fact to mind because of its almost entire absence from the teaching and literature of the day which is put forth as homœopathic in our colleges, and so generally in the writings for our journals. The prevailing effort is, in these last days, to substitute for this a *generalization,* which may relieve the prescriber from the careful toil of dealing, as the law requires, with what may seem to him, at times, as only, or no better than, "a wilderness of symptoms," though so to deal with them alone constitutes a practice *homœopathic.* The modern resort to *generalization* in pretended homœopathic practice and teaching is ever an exclusion of all which is essential to the philosophy of the natural law of therapeutics.* This, for its practical administration, requires a gathering and analysis of *all* the elements of the sickness to be cured, before any other step of clinical duty can be taken under the guidance of this law. The generalizer thrusts this requirement of law aside, takes the sickness as a whole, a *thing* with a *name* to it, and then, if he has any regard to our law, or is presumed to have, he further generalizes that a certain drug, or it may be *drugs* (if he be an alternate), has produced in the healthy organism a similar *thing.* He *names* his *thing,* and is thus found on ground wholly allopathic, or nearly so, and that he obtains his medicines from *Smith* or *Tafel,* and gives them in small or large doses, does not change this fact in the least.

We can see but one excuse for this abandonment of the philosophy of the natural law of therapeutics. It is certainly *easier* to say before a given "wilderness of symptoms"—"*ague,*" than to analyze and individualize each member of this "wilderness," and far easier, before this name, "*ague,*" to say "*quinine*" than to hunt the materia medica for a recond of a similar "wilderness." But then this whole proceding is only a masking of *old-school* physics behind the pretense of the *homœopathic* name.

This generalization can never be a resort of the truly *specific* prescriber. It may be "*easy*" for the *prescriber,* but how is it for the *sick?* Because it violates natural law, it cannot be safe, and can only be successful when the prescriber's *guessing* is better

---

* This exclusion appears to have been resorted to, and this generalization substituted, in the interest of the lazy or the ignorant. It is a resort of the endeavor to make that *easy* which in the nature of the case can *never* be made easy. The result is only a sacrifice of the truth.

than his false *principles—i. e.*, it can only be successful by a *lucky* blunder.

In carrying out our purpose we have attempted to give, in a part of our paper, a specimen of simple differentiation of the elements of the action of these drugs on certain organs and functions. As to other organs and functions, we have given the record of each close to the other, and then have given a differentiation of these groups to show how this essential process of all true study of our materia medica, and all true practice with it, should be carried on. A part of the record, as stated above, has been given, and the differentiation has been left for the student to make for himself, he having been shown how this work is to be done. As a further help to him in this work, the differentiating elements of this last portion of the record have been marked by putting them in italics. The paper, as a whole, is only given as a specimen of a method of *true* study of materia medica.

We were some years ago an observer of an instance of the generalization we have spoken of, when a candidate for the degree of Doctor of Medicine presented himself before the Professor of Materia Medica of the College from which he had recently been receiving instruction, that he might be examined by this functionary as to his knowledge of this most beautiful and useful science. I was all attention. I wanted to see just how the good man would proceed in drawing out the evidence that this candidate possessed such knowledge of this science as would convince the examiner that, so far as this was concerned, he might be safely certified to as competent to administer it at the bedside of the sick. What was the *kind* of evidence this Professor would seek, for his satisfaction, that this candidate was duly qualified in this science to enter on the practical duties of a healer of the sick? His first question to the candidate was enlightening as to the *kind* of evidence he was after, and is also illustrative of his own unfitness to teach this science, and therefore unfitness to certify to another's knowledge of it.

This was the Professor's first question to the candidate: "Will you please to tell me, sir, the difference between the action of Aconite and Belladonna?" Well, here is a poser for him, thought I. And so it turned out, for the candidate could only reply, "I don't know." Of course he didn't so know as to give the short, didactic answer the Professor looked for. He wanted, in a few words, what it has taken all these many pages to express as to the many, *many* differences in the action of these drugs. Here was a *stick* in the very beginning, and the effort of the Professor to lift the candidate over it was farther illustrative of the Professor's ideas of materia medica and presumably of how he taught it. This is how he did this, by asking another question: "Is it not, sir, that Aconite acts more on the *organs of circulation*, and Belladonna on the *nervous system?*" The candidate said "Yes." This question did not suggest a truth, and the answer yielded to a temptation and assented to a falsehood.

Both these remedies act on the circulation and both on the nervous system. It is *not* that *either* acts on the one more than on the other, but that each acts on *both*, and each on the one or the other of these two systems in its own fashion, and that this is *different* from the fashion of the other. The Professor had no conception of our science of materia medica as before this essential element of knowledge necessary to its successful administration in clinical duties, viz.: the *differentiation* of the *elements* of drug action. It is not difficult, therefore, to forecast the character of his attempts to teach classes what he would have them accept as our *science* of materia medica. He presented to them, in a very poor manner, a very poor imitation of *old-school generalization*, and this was to pass for a science wholly made up of *individual* facts. We ask, Is this very different from the way in which this science is disposed of in our colleges generally? And further, Do the graduates they turn out know when they leave the class-room that they go out not having been taught the *homœopathic* materia medica at all? Do they know, these graduates, that as to a knowledge of the true character of our materia medica, they do not carry away from their class-room enough to constitute them even decent caricatures of that which their received diplomas certify they have been made by the instruction of their colleges, *viz.: doctors?* It is plain to any one who is conversant with the history they have made for the American Institute, the last few years, that they do not know, for apparently they are not in the least ashamed of their disgraceful work. They do not know enough of *true* Homœopathy to know that this is a work to be ashamed of. Now, who is responsible for this ignorance? Who, indeed, but these colleges who have sent them forth with certificates, which as to these graduates and the world are only false witnesses as to their possession of qualifications for the practical life and duties of homœopathic healers.

---

We are authorized by the publishers, Messrs. Gross & Delbridge, homœopathic pharmacists, of Chicago, to announce that the third edition of Cowperthwaite's *Text-Book of Materia Medica* will be issued about the 1st of September. It will be much enlarged and improved.

